# Clinical Application of the Novel Cell-Based Biosensor for the Ultra-Rapid Detection of the SARS-CoV-2 S1 Spike Protein Antigen: A Practical Approach

**DOI:** 10.3390/bios11070224

**Published:** 2021-07-06

**Authors:** Sophie Mavrikou, Vasileios Tsekouras, Kyriaki Hatziagapiou, Foteini Paradeisi, Petros Bakakos, Athanasios Michos, Antonia Koutsoukou, Elissavet Konstantellou, George I. Lambrou, Eleni Koniari, Elizabeth-Barbara Tatsi, Joseph Papaparaskevas, Dimitrios Iliopoulos, George P. Chrousos, Spyridon Kintzios

**Affiliations:** 1 Laboratory of Cell Technology, Department of Biotechnology, Agricultural University of Athens, EU-CONEXUS European University, 11855 Athens, Greece; tsekouras@aua.gr (V.T.); stud315079@aua.gr (F.P.); 2 First Department of Pediatrics, National and Kapodistrian University of Athens, “Aghia Sophia” Children’s Hospital, Thivon 1, 11527 Athens, Greece; khatziag@med.uoa.gr (K.H.); amichos@med.uoa.gr (A.M.); glamprou@med.uoa.gr (G.I.L.); 3 Physiotherapy Department and Department of Nursing, Faculty of Health and Care Sciences, State University of West Attica, Agiou Spiridonos 28, 12243 Egaleo, Athens, Greece; 4 First University Department of Respiratory Medicine, “Sotiria” Hospital, Medical School, National and Kapodistrian University of Athens, 152 Mesogeion Ave, 11527 Athens, Greece; pbakakos@med.uoa.gr (P.B.); akoutsou@med.uoa.gr (A.K.); eliskonst@yahoo.gr (E.K.); 5 University Research Institute of Maternal and Child Health and Precision Medicine, and UNESCO Chair on Adolescent Health Care, National and Kapodistrian University of Athens, “Aghia Sophia” Children’s Hospital, Thivon & Livadias 8 str, 11527 Athens, Greece; eltheo1983@gmail.com (E.K.); etatsi@med.uoa.gr (E.-B.T.); chrousos@gmail.com (G.P.C.); 6 Department of Microbiology, Medical School, National and Kapodistrian University of Athens, Mikras Asias 75, 11527 Athens, Greece; ipapapar@med.uoa.gr; 7 Emergency Operations Center, National Public Health Organization (NPHO), Agrafon 3-5, 15123 Athens, Greece; d.iliopoulos@eody.gov.gr

**Keywords:** Bioelectric Recognition Assay (BERA), membrane engineering, public health surveillance, Point-of-Care (POC), S1 spike protein, rapid antigen test, screening, serological assay, severe acute respiratory syndrome-coronavirus 2 (SARS-CoV-2)

## Abstract

The availability of antigen tests for SARS-CoV-2 represents a major step for the mass surveillance of the incidence of infection, especially regarding COVID-19 asymptomatic and/or early-stage patients. Recently, we reported the development of a Bioelectric Recognition Assay-based biosensor able to detect the SARS-CoV-2 S1 spike protein expressed on the surface of the virus in just three minutes, with high sensitivity and selectivity. The working principle was established by measuring the change of the electric potential of membrane-engineered mammalian cells bearing the human chimeric spike S1 antibody after attachment of the respective viral protein. In the present study, we applied the novel biosensor to patient-derived nasopharyngeal samples in a clinical set-up, with absolutely no sample pretreatment. More importantly, membrane-engineered cells were pre-immobilized in a proprietary biomatrix, thus enabling their long-term preservation prior to use as well as significantly increasing their ease-of-handle as test consumables. The plug-and-apply novel biosensor was able to detect the virus in positive samples with a 92.8% success rate compared to RT-PCR. No false negative results were recorded. These findings demonstrate the potential applicability of the biosensor for the early, routine mass screening of SARS-CoV-2 on a scale not yet realized.

## 1. Introduction

Assays targeting antigenic moieties of the respiratory syndrome-coronavirus 2 (SARS-CoV-2) have been recognized as one of the most promising approaches to successfully identify asymptomatic carriers of the virus, especially during the first two weeks following infection [1,2,3] and taking into consideration the fact that asymptomatic infection rates range globally between 18% and 42% [4]. In practical terms, screening for viral antigens differentiates itself from antibody-targeting serology tests since it does not depend on host antibody accumulation to detectable levels in the second and third week of illness [5]. The interest in screening individuals for COVID-19 infection in point-of-care (POC) settings is concomitant with the wide vaccination against the virus [6]. Although nucleic acid-based tests are considered the golden standard for SARS-CoV-2, they are prone to false-negative or even contradictory results, especially before or at symptom onset [7,8,9]. In the context of immunological assays, antigen-detecting methods are advantageous over serology-based ones, since no prior seroconversion (usually peaking around one to two weeks after the onset of symptoms) is required [10,11]. Among the four major surface protein types, spike proteins have a prominent role in the development of diagnostic assays, since they are directly involved in the mechanism of host cell entry through their ability to bind to the angiotensin-converting enzyme 2 (ACE2), in turn determining the level of infectiousness and virulence of the virus. More significantly, although the most abundant protein of the virus is the nucleocapsid one (N), spike (S) proteins, being the host attachment ones, are considered more specific [12].

Currently, the overwhelming majority of antigen rapid tests for SARS-CoV-2 are targeting the nucleocapsid protein, usually coupled with lateral flow assay configurations [13]. They are usually characterized by high (>90%) specificity and clinical accuracy [14]; however, they suffer from poor sensitivity, at least compared to RT-PCR (Real-Time Polymerase Chain Reaction) [15]. For this reason, they are mainly used in symptomatic patients [16,17]. On the other hand, a more promising target for antigen assays is the S1 spike protein of SARS-CoV-2, since it is centrally involved in the early stages of infection and, thus, is the major antigen recognized by humoral and cellular immune responses as a mirror of the whole virus [18,19], as well as a molecular tool to discriminate between different coronaviruses (e.g., SARS-CoV-2 vs. SARS-CoV) [20].

In spite of its attractiveness as a basis for the development of coronavirus antigen tests, extremely few reports are available on the sensitive and reliable detection of the SARS-CoV S1 protein [21,22,23]. Even more rare are reports on clinical validation and/or practical application of such approaches [24].

Our team has recently reported the proof-of-concept development of a novel biosensor for the ultra-rapid (3 min) and sensitive detection of the SARS-CoV-2 S1 spike protein [25], with a limit of detection of 1 fg/mL and a semilinear range of response between 10 fg and 1 μg/mL. The biosensor was based on mammalian cells, which were engineered by electroinserting the human chimeric spike S1 antibody. According to a well-established process known as Molecular Identification through Membrane Engineering [26,27,28], binding of the SARS-CoV-2 S1 protein to complementary membrane-based antibodies resulted in a considerable and selective change in the membrane-engineered cell bioelectric properties, which was measured with a customized portable read-out device operated via smartphone/tablet [29,30]. This is a broad methodology based on the electroinsertion of antibody molecules on the cell surface of mammalian cells. The attachment of a specific antigen to its respective antibody causes a measurable change in the cell membrane structure, leading to a change in the cell membrane potential. Moreover, membrane-engineered cells have been utilized as biorecognition elements in various sensors applied for the detection of human viruses such as Hepatitis B Virus [31], Hepatitis C Virus [29], SARS-CoV-2 [25,32], as well as several plant viruses such as Cucumber mosaic virus (CMV) [28], Potato virus Y (PVY), and Tobacco rattle virus (TRV) [33]. Very recently, our system has been validated by another independent research team on 110 positive and 136 negative SARS-CoV-2 samples tested by RT-PCR [32]. A total sensitivity of 92.7% and a specificity of 97.8% was demonstrated. However, it should be emphasized that samples assayed with the biosensor were processed according to the specifications for molecular (RT-PCR) testing, i.e., the samples were considerably pretreated before testing.

In the present study, we report on the first clinical application of an improved version of our novel biosensor, configured as ready-to-use. Positive patient-derived samples were identified with a 92.8% score compared to RT-PCR, while no false-negative results were recorded. We demonstrated that, following further validation, our approach could be applied for early and routine population testing for SARS-CoV-2 with minimal sample processing, easy use, and at a scale of application not realized so far.

## 2. Materials and Methods

### 2.1. Cell Culture Conditions

The SK-N-SH neuroblastoma cells (ATCC^®^ HTB-11™) were cultured under standard conditions (37 °C, 5% CO_2_), in 1x Minimum Essential Medium (MEM) with Earle’s balanced salt (Biowest, Nuaillé, France). Fetal bovine serum (FBS) (10%) (Thermo Fisher Scientific, Waltham, MA, USA) was added to the culture medium, as well as 2 mM of l-glutamine, 0.1 mM nonessential amino acids, 1.0 mM sodium pyruvate (Biowest, Nuaillé, France) and 1 U μg^−1^ antibiotics (penicillin/streptomycin). Cells were subcultured once or twice per week in a 1:10 ratio. Trypsin–EDTA (0.05% trypsin, 0.02% EDTA) (Biowest, Nuaillé, France) was used for cell dissociation from the culture flasks, after treatment for 3–10 min.

### 2.2. Sensor Fabrication from Membrane-Engineered Cells (SK-N-SH/Anti S1)

Membrane engineered cells were fabricated after the electroinsertion of SARS-CoV-2 Spike S1 antibody (Recombinant Anti-SARS-CoV-2 Spike Glycoprotein S1 antibody [CR3022]—Chimeric, Cambridge, United Kingdom) into the cell membrane, according to the prior publication of Mavrikou et al. [25]. The SK-N-SH cells were harvested from a culture vessel by trypsinization. Cells were resuspended in phosphate-buffered saline (PBS) (pH 7.4), at a final density of 2.5 × 10^6^/mL, alongside the antibody (0.5 μg/mL) and maintained at 4 °C for 20 min. The cell suspension was then subjected to electroporation with two pulses of an electric field at 1800 V/cm (Eppendorf Eporator, Eppendorf AG, Hamburg, Germany) and was immediately transferred in a Petri dish (60 × 15 mm) enriched with cell culture medium and placed in the incubator overnight.

**Figure 1:**
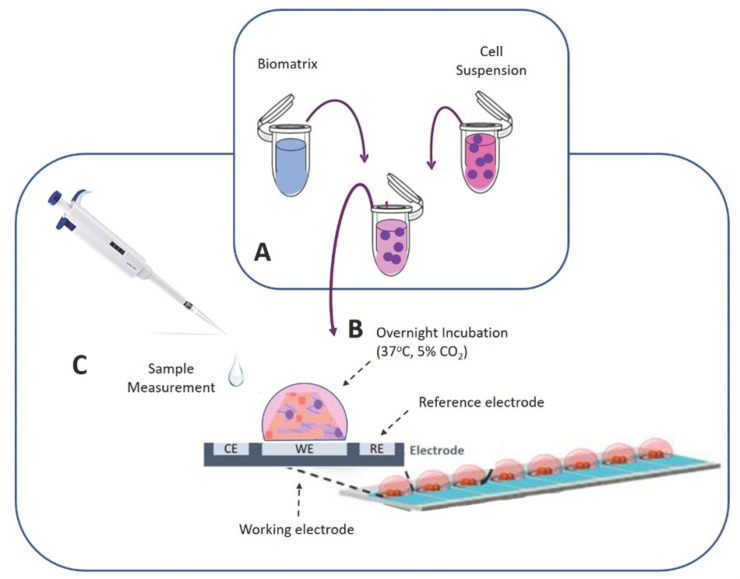
Cells were mixed with the hydrogel matrix (**A**) and were placed onto the working electrode’s surface. The electrodes were put in petri dishes and were incubated overnight at 37 °C, 5% CO_2_ (**B**). The next day the modified electrodes with the 3D cultures were ready for use (**C**).

The next day, the membrane-engineered cells were detached from the petri dish with pipetting and collected with PBS (pH 7.4) in Eppendorf tubes in desirable concentrations. The interactions of membrane-engineered cells with the spike S1 protein were then recorded either in cell suspensions (50 × 10^3^ cells per sensor) or in three-dimensional (3D) cell cultures (25 × 10^3^ cells per sensor). In the case of 3D cultures, a custom-made extracellular collagen-based (at least 0.5% *w/v*) hydrogel matrix was used for cell immobilization (the formulation is subjected to patent submission) (Figure 1).

### 2.3. Bioelectric Real-Time Measurements: Biosensor Set-Up and Experimental Design

It is well documented that the alterations of the membrane potential of membrane-engineered cells due to the interactions between the electroinserted receptor molecules and analyte anions, cause the production of electric signals that can be recorded, according to the principle of the Bioelectric Recognition Assay (BERA) [29,30]. Our method of choice for measuring potential changes was the determination of Open Circuit Potential (OCP) values. OCP is the potential established between the working electrode and the environment, with respect to a reference electrode, which will be placed in the electrolyte close to the working electrode. OCP is a passive method, meaning that the counter electrode (necessary to pass current through the cell) circuitry of the potentiostat is bypassed. In this mode, only the resting potential between reference and working electrode is measured [34].

**Figure 2:**
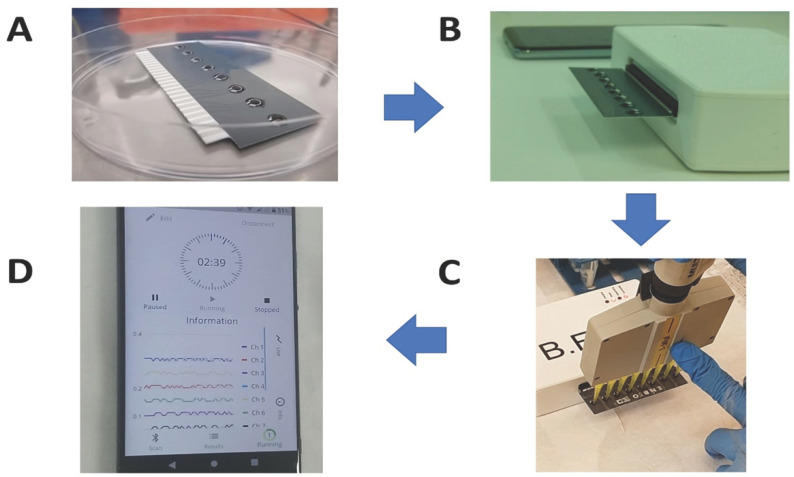
Experimental set-up of the membrane-engineered cell-based biosensor’s assembly. Εight-channel carbon screen-printed electrodes were prepared with custom-made hydrogel-immobilized membrane-engineered cells (**A**). The electrode containing the 3D cell cultures was placed to the measurement device (**B**). Each sample was applied (8×) to the testing positions (cell-covered electrodes) while the electrode strip was connected to a bespoke portable potentiometer which is connected to a tablet device for the recording of the measurements immediately after the sample application (**C**). The electric signal is continuously visualized through a voltage vs. time graph on the screen of a smartphone connected via Bluetooth to the device (**D**).

For this purpose, a customized potentiometer with an eight-channel configuration was used to record the membrane-engineered cells’ electric properties. Carbon screen printed multichannel electrodes were used (working electrode: carbon, reference: Ag/AgCl) on a disposable sensor strip (iMiCROQ S.L., Tarragona, Spain) (Figure 2A,B).

Samples (10 μL) were added to the top of each carbon electrode containing membrane engineered cells either in suspension or 3D culture by using a multichannel automatic pipette (Figure 2C). Upon the addition of the sample, a time series of cell responses was recorded for 180 s by potentiometric measurements in volts (360 values, sampling rate 2 Hz). The recorded measurements were uploaded via a tablet/Bluetooth communication to a cloud server and further analyzed (Figure 2D) [35].

Each sample was tested eight times using a set of eight individual sensors and each experiment was performed in triplicate, while each experimental set was repeated at three different dates (*n* = 24). The biosensor measurements are expressed as normalized responses according to the following equation:

(1) $$\text{Normalized Biosensor Response}\;=\;\frac{\text{Control}-\text{Sample}}{\text{Control}}$$

The results are expressed as mean ± SEM, whereas the differences between the means were tested for statistical significance using an analysis of variance and Student’s *t*-test.

The present study was carried out in three experimental steps. Initially, we investigated our system’s lower limits of detection. For this purpose, we spiked SARS-CoV-2 negative patient nasopharyngeal samples with several low concentrations of SARS-CoV-2 spike S1 protein (2 fg/mL, 20 fg/mL, 200 fg/mL, 2 pg/mL, 20 pg/mL, 200 pg/mL, and 2 ng/mL) to reflect the usual concentrations of viral protein ranging from asymptomatic to moderately ill patients in vivo. Then, we tested the spiked samples with the membrane engineered cells in suspension or in 3D culture.

In the second experimental step, we evaluated the BERA biosensor based on 3D membrane-engineered cell culture with SARS-CoV-2 negative and positive patient nasopharyngeal samples. Finally, in the third experimental step, cell viability in 3D cultures was assessed for long-term storage as part of the biosensors’ consumable components.

### 2.4. Viability Monitoring of Membrane-Engineered Cells in 3D Extracellular Immobilization Matrix

To access the proliferation and viability of the cell membrane engineered with the SARS-CoV-2 Spike S1 antibody in the hydrogel immobilization matrix, we microscopically observed cell viability for two weeks within three days intervals. The Trypan Blue Exclusion Assay was performed in order to determine cell death [36]. Cell viability changes were captured (from Day 1 = 24 h after electroporation) by an inverted microscope (ZEISS Axio Vert.A1, Carl Zeiss Microscopy, LLC, White Plains, NY, USA), and the pictures were processed by ZEN lite and ImageJ software.

### 2.5. Patient Recruitment, Clinical Examination and Specimen Collection

All patients included in the study had a positive RT-PCR test on nasopharyngeal swabs for SARS-CoV-2 at the time of hospital admission and prior to sample collection for antigen detection with the biosensor. Overall, the severity of their disease was mild to moderate, except for one patient who was finally intubated. Nasopharyngeal swabs for antigen detection were collected, following a standardized procedure, using Dacron swabs (FIRATMED, 8870000244, Albacete, Spain). Fourteen patients and fourteen healthy volunteers consented to have two replicate specimens collected. Healthy volunteers had already received two doses of COVID-19 mRNA vaccine and were matched to patients. Matching parameters included sex, age, and medical history. All specimens for antigen testing were collected at 12 March 2021. Each specimen was transported in a sterile centrifuge tube (Greiner 15 mL centrifuge tube). For each patient or volunteer, the first swab was placed into 10 mL of sterile 0.9% saline solution and the second one into 10 mL of PBS at pH 7.4 (Gibco PBS, 10010023, Thermo Fisher Scientific, Waltham, MA, USA). Specimens were stored on ice for transport to the clinical laboratory for testing. Upon receipt, the specimens were stored at –80 °C, until the process [37,38].

The clinical characteristics of patients with COVID-19 as well as their medical history are described in Supplementary Table S1 of the supporting information.

## 3. Results

In a previous study, we demonstrated the proof-of-concept detection of the SARS-CoV-2 spike S1 protein by means of a Bioelectric Recognition Assay recording the alterations of the cellular bioelectric properties of membrane-engineered mammalian cells bearing the human chimeric spike S1 antibody [25]. As a next step, in the present investigation we tried to evolve our technology: (i) using new cellular biorecognition elements to augment the biosensor’s sensitivity, (ii) by the integration of the biorecognition elements into a collagen-like hydrogel for increasing storage time, and (iii) by the evaluation of the assay’s performance with the use of clinical samples.

### 3.1. Membrane-Engineered SK-N-SH/Anti-S1 Cells Have a Distinct Response against the SARS-CoV-2 Spike S1 Protein in Suspension and 3D Culture Conditions

**Figure 3:**
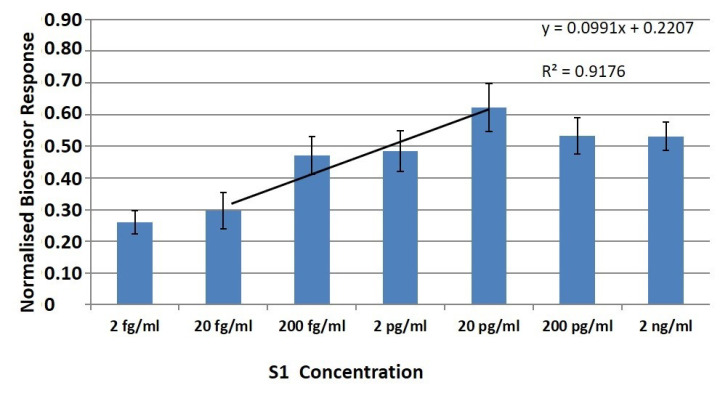
Normalized (vs. control) cell biosensor’s responses in suspension against the SARS-CoV-2 spike S1 protein. SK-N-SH/anti-S1 cells membrane-engineered with 0.5 μg/mL of human monoclonal antibodies were used as the biorecognition elements. Results (mean ± SE) are presented after three minutes (columns) of sample–cell interaction. Results are expressed as normalized biosensor responses ([control-sample response]/control, *n* = 24).

**Figure 4:**
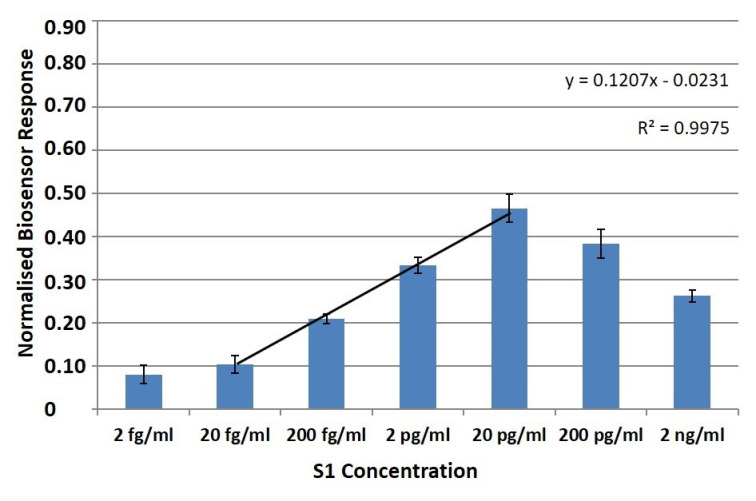
Normalized (vs. control) 3D immobilized cell biosensor’s response to the SARS-CoV-2 spike S1 protein. SK-N-SH/anti-S1 cells membrane engineered with 0.5 μg/mL of human monoclonal antibodies, were used as the biorecognition element. Results are presented after three minutes (columns) of sample–cell interaction. Results are expressed as normalized biosensor responses ([control–sample response]/control, *n* = 24).

Membrane-engineered cells with 0.5 μg/mL human monoclonal anti-S1 antibodies were exposed to increasing concentrations (2 fg/ML–2 ng/mL) of the SARS-CoV-2 spiked S1 protein. A clearly observable and very rapid response was produced, measurable within 3 min of cell-protein interaction. The generated signal from the membrane-engineered cells cultured either in suspension (Figure 3) or in the 3D extracellular matrix (Figure 4) was clearly distinguishable.

A 10 μL sample volume of various concentrations of the S1 protein was administered to a population of 25 × 10^3^ membrane engineered cells. Measurements at each S1 protein concentration were distinctly and significantly different from the control solutions (i.e., zero S1 concentration). Results were quite reproducible, with a very low limit of detection (LOD) at two (2) fg/mL.

A concentration-dependent linear pattern was observed after the administration of increasing concentrations of the SARS-CoV-2 spike S1 protein, in the range of 2 fg–20 pg/mL. Additionally, the responses of the cells in 3D cultures as well as in suspension, upon the addition of the S1 protein solution, were similar. The 3D cultured cells produced more reproducible responses for each applied concentration of S1 protein, in comparison with the respective responses of the cell suspensions. This conclusion is based on the relative standard error comparison (RSE), since in all assayed concentrations that are equal or higher than 20 fg/mL, the RSEs of the normalized 3D cultured cells’ responses (average RSE: ±9.4%) were lower than those obtained from the cells in suspension (average RSE: ±14.3%).

### 3.2. Ultra-Rapid Detection of the SARS-CoV-2 S1 Spike Protein Antigen in Clinical Samples by the Membrane-Engineered SK-N-SH/Anti-S1 Cells in Suspension and 3D Conditions in Comparison with RT-PCR Results

Two different approaches were applied, based on membrane engineered cells in suspension and in 3D cultures. Comparison of the performances of suspension and 3D cultures to those of RT-PCR with samples from all categories of patients gave similar results regardless of the methodology used. In total, 14 (58.33%) of the 24 samples were positive for SARS-CoV-2 virus. Our cell-based biosensor successfully detected the virus in 13 out of 14 specimens (92.8%) confirmed to be positive by RT-PCR. In addition, no false-positive results were observed in the case of negative samples (*n* = 10).

**Figure 5:**
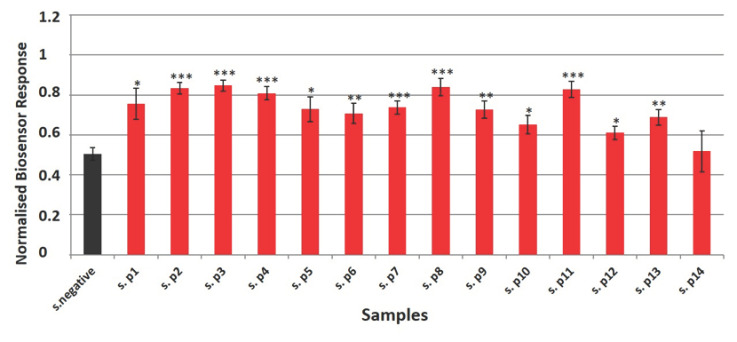
Normalized cell biosensor’s responses to clinical samples from patients (*n* = 14) and healthy donors (mean of 10 samples—black column). The membrane engineered cells with the Anti-SARS-CoV-2 Spike S1 antibody were added onto the electrode’s surface in suspension. Results are presented after three minutes of sample–cell interaction. Results are expressed as normalized biosensor responses ([control–sample response]/control). Significant differences (Student’s *T*-test) between normalized biosensor responses * < 0.05, ** < 0.01, *** < 0.001.

**Figure 6:**
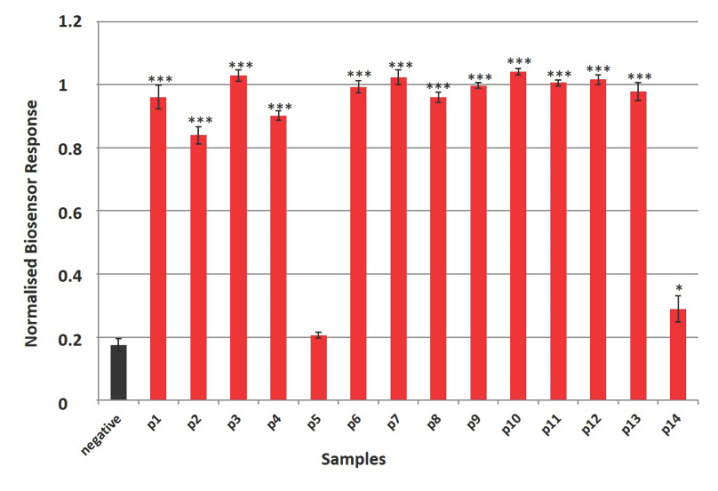
Normalized 3D cell biosensor’s responses against clinical samples from patients (*n* = 14) and healthy donors (mean of 10 samples—black column). Results are presented after three minutes of sample–cell interaction. Results are expressed as normalized biosensor responses ([control–sample response]/control). Significant differences (Student’s *T*-test) between normalized biosensor responses * < 0.05, ** < 0.01, *** < 0.001.

As can be seen in Figure 5, the normalized cell biosensor’s responses derived from membrane engineered cells in suspension successfully identified six specimens (42.8%) with statistical significance *p* < 0.001, three specimens (21.4%) with statistical significance *p* < 0.01 and four specimens (28.5%) with statistical significance *p* < 0.05. Non- statistically significant results were observed in the case of specimen 14. When we used cells immobilized in a hydrogel-based extracellular matrix (Figure 6), the biosensor identified 12 specimens (85.7%) with statistical significance *p* < 0.001 and one specimen (7.1%) with statistical significance *p* < 0.05. In this case, non-statistically significant results were observed for specimen 5.

### 3.3. Membrane Engineered Cells Can Be Maintained in the Hydrogel-Based Matrix for a Minimum Two Week Interval

**Figure 7:**
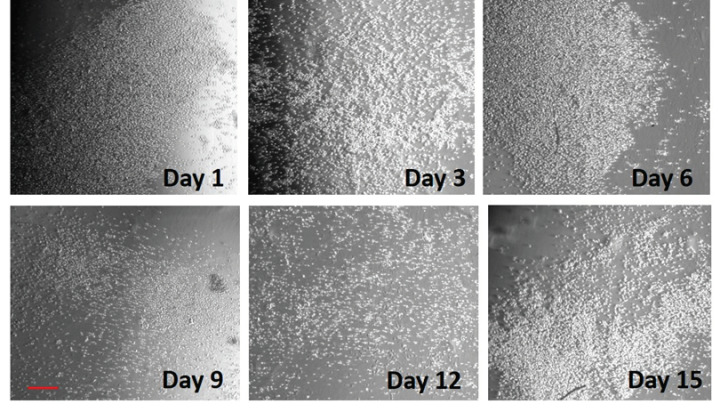
Panoramic view of membrane-engineered cells cultured in the hydrogel matrix after incubation for 15 days. Pictures were taken every 3 days. Scale bars = 50 μm.

**Figure 8:**
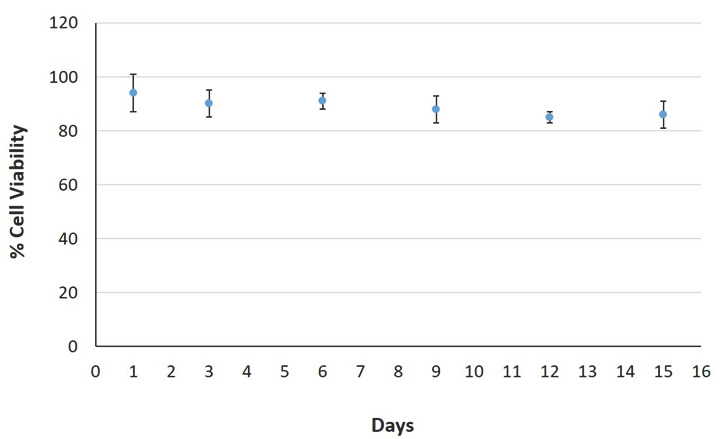
The graph depicts the percentage of cell viability during time.

The day after electroporation, cells were detached from the Petri dish and transferred to the hydrogel extracellular matrix. The assessment of cell proliferation started after 24 h. Fresh medium was added every two days. Pictures of the cells were taken every 3 days. Figure 7 shows the microscopic observations of the 3D cultures after 15 days of incubation in a cell culture chamber, whereas Figure 8 depicts the graphical representation of cell viability as the percentage of live cells of the total number of cells. As can be seen, the immobilization matrix did not seem to negatively affect cell viability up to day 15. This also coincided with the highly reproducible biosensor responses against clinical samples throughout the observation period.

## 4. Discussion

Due to the brief history of the COVID-19 epidemic and in spite of its global dimension, diagnostics of the disease are still evolving. RT-PCR tests on nasopharyngeal and throat swabs are considered the current golden standard for the reliable identification of positive samples, although problems related to the costs of mass application, occasional false-negative results and lack of detection of the virus during the early phase of the infection [1]. Another drawback of molecular testing is the fact that detection of viral RNA does not always equal a viable virus [7]. On the other hand, the focus of antigen tests is steadily increasing thanks to a number of clinical trials demonstrating their high level of specificity and sensitivity, and a high agreement rate with RT-PCR (depending on the viral load) at least as far as the nucleocapsid protein is concerned [3,39,40,41]. An additional advantage of antigen assays is their lower variability since they are independent of aberrant changes in viral load per stage of infection [42]. Furthermore, antigen-based tests are superior to SARS-CoV-2 serology because they avoid the pitfall of false-negative results due to possible weak host immune responses [43]. That said, several challenges are still associated with the achievement of a reliable testing process, for example, minimizing the variation of results between different types of samples as well as the timing of sample collection relative to the onset of the illness and the method of sample collection [44]. Antigen tests are advantageous in this respect, too, since viral RNAs are much less stable during transport and storage than proteins.

As already mentioned in the Introduction, there are very few antigen-based assays for the detection of spike SARS-CoV-2 S1 antigen that have been clinically tested so far. For example, Lee et al. [24] very recently reported the development of a lateral flow immunoassay based on the S1-binding SARS-CoV-2 receptor ACE2 with a limit of detection of 1.86 × 10^5^ copies/mL. Their method was clinically validated on nasopharyngeal swab samples from four COVID-19 patients and four healthy subjects. The subsequent analysis showed three positive results from confirmed clinical specimens with RT-PCR analysis, while no false-positive results were recorded. Our cellular biosensor-based approach is the second example of a clinically validated method in the rapidly expanding field of technology targeting coronavirus spike proteins as a strategy for global surveillance of COVID-19 and diseases possibly related to other respiratory viruses.

One of the major challenges for the broad application of cell-based assays (CBAs) in routine analytical and diagnostic needs is their potential for practical applicability, often limited by specific requirements for the availability of custom cell cultures (as the primary consumable) and associated equipment at the site of the assay/testing process. This condition renders the wide use of cell-based biosensor principles practically impossible, especially in resource-limited environments. Overcoming the hurdle of limited cell viability by using specific cell types (e.g., fish gills) or integrating microfluidic/organ-on-chip circuits in the consumable modules of biosensing platforms is only one of the potential areas for improving the performance of advanced CBAs [45,46,47]. Equally important is the ability to achieve highly reproducible test results within the linear range of responses; this is not always guaranteed when using cell suspension cultures, in particular when cell densities are not kept constant or are not clearly defined. For example, a common problem for cells removed from optimal incubation conditions (e.g., 37 °C, 5% CO_2_) to be used under field conditions is their gradual loss of viability over a period of hours or days, which in turn may lead to major inconsistencies of their response to a certain analyte. Finally, handling cell cultures at the testing site demands the engagement of technically skilled operators and can be time-consuming.

In the present study, we improved our previously developed [25] and clinically validated [30] biosensor for the detection of the SARS-CoV-2 S1 spike antigen giving emphasis on the following priorities: (i) increasing the practical ease of application of the novel system in view of its potential large scale use; (ii) ensuring the derivation of reproducible, cell batch-independent test results; and (iii) reducing the duration of each assay as well as the time between subsequent assays, as an additional measure to reach a high throughput performance. Our major intervention to achieve these goals was the use of a cell immobilization approach. This not only enabled the vertical extension of cell viability (at least up to two weeks) (Figure 7 and Figure 8) but also the considerable increase of ease of use and speed of each test, with the lag period between subsequent assays being reduced to just a few seconds and practically corresponding to the time required for changing the cartridge containing the electrode strip of the 3D-immobilized cells. It is also worth mentioning that the consumable cartridges were batch stored during the whole experimental period with no impact on the reproducibility of the test results. It has been previously reported that cell immobilization is one of the most favorable methods for increasing cell viability [48,49] (up to a few weeks [50]), an approach that has also been successfully applied on membrane-engineered mammalian cells [51].

Our results indicate that as concentrations of analyte increase, the signal from immunoassays increases as well; thus, the proposed biosensor can quantitatively determine the S1 protein to concentrations up to 20 pg/mL (especially in the case of the 3D cultures). The increase in the response should be linear in accordance with the increase of the analyte’s concentration. However, as the concentration of analyte increases above a certain point, the system gets saturated and the signal begins to decline. This phenomenon is known as the hook effect [52]. In this case, a concentration-dependent linear pattern was observed after the administration of increasing concentrations of the SARS-CoV-2 spike S1 protein, in the range of 2 fg/mL–20 pg/mL. Additionally, the responses of the cells in 3D cultures as well as in suspension, upon the addition of the S1 protein solution, were similar. The 3D cultured cells produced more reproducible responses for each applied concentration of S1 protein, in comparison with the respective responses of the cell suspensions.

On the performance side, the biosensor was able to successfully identify positive samples with 93% accuracy, while no false negative samples were recorded. Our results are in accordance with a previous study with the BERA method conducted by Apostolou et al. [32]. The biosensor showed a sensitivity of 92.7% (102/110) against RT-PCR with clinical samples. In our case, a rather remarkable reproducibility (average RSE%: 3.6%) was determined, once again demonstrating the merits of the cell immobilization approach when compared to cells in suspension (average RSE%: 6.8%).

The selection of clinical samples was based on matching factors, such as age and sex, that are commonly used in clinical studies. This process was performed in order to improve study efficiency by generating a comparable group of negative samples selected from the same population as the positive cases [53]. As can be seen in Table S1, patients 2, 7, 8, 10, 11, and 12 had a positive RT-PCR test a few days prior or after specimen selection. Furthermore, patients 1, 4, 6, 9, and 11 had been positive for SARS-CoV-2 almost one month prior to the sample collection, indicating that the biosensor (with both cell culture approaches) is able to detect the disease even at very low concentrations of the virus. Even though patient 5 was initially found positive for COVID-19 at 20 February 2021, the second RT-PCR test performed at 17 March 2021, five days after specimen selection for antigen detection, was negative. The lack of viral copies might be a possible explanation for the ambiguous results of both cell-based biosensor systems used in the study (Figure 5 and Figure 6). Moreover, the biosensor using cell suspensions as the bioelectric recognition part was not able to identify as positive for SARS-CoV-2 specimen collected from patient 14. This result could be attributed to the biosensor’s interference with the complicated medical treatment he was receiving, indicating once again the value of cell immobilization (*p* < 0.05).

Based on our current laboratory-scale protocol for manufacturing consumable cell-bearing electrodes containing the cellular biorecognition material and the electrode interface, as well as our extended experience with commercial applications of membrane-engineered cells, we have calculated a capacity of at least 1000 test kits each day from a small production line operated by a single person. Furthermore, immobilization of cells at a much lower density compared to the previously reported cell suspension-based approach [24] is directly associated with a drastic reduction of the consumable costs. To our best knowledge from current collaborations with national and international partners, consumable cartridges containing electrodes with immobilized cells can be shipped by common methods of transport to remote end-users with no loss in functionality for at least 2–3 weeks.

## 5. Conclusions

In this report, we demonstrated the clinical applicability of a practically improved and readily employable version of our previously published cell-based biosensor for the detection of COVID-19 in nasopharyngeal swabs. Since the completion of the proof-of-concept development of the original assay, we have launched an optimization process including the expansion of the number of cell lines to be membrane-engineered with the human chimeric spike S1 antibody and by further investigating the cross-reactivity and specificity of the biosensor, in particular against the S proteins of other coronaviruses. In addition, among our immediate next research goals is the development, using our methodological approach, of a biosensor for the detection of the NC antigen and its subsequent application in saliva samples together with the present S1-specific biosensor. In this way, we expect that both the sensitivity and the selectivity of the assay will be increased [9].

## Data Availability

The data presented in this study are available on request from the corresponding author. The data are not publicly available due to medical confidentiality and privacy.

## Supplementary Materials

The following are available online at https://www.mdpi.com/article/10.3390/bios11070224/s1, Table S1: The timeline of patients’ personal and medical history.
