# Supplementary material for: Clinical Application of the Novel Cell-Based Biosensor for the Ultra-Rapid Detection of the SARS-CoV-2 S1 Spike Protein Antigen: A Practical Approach
Source: Biosensors (Basel). 2021 Jul 6;11(7):224. doi: 10.3390/bios11070224 (PMC8301797; doi:10.3390/bios11070224)
Supplement: Supplementary file 1 [file biosensors-11-00224-s001.zip › biosensors-1264758-supplementary.pdf]

Table S1. The timeline of patients' personal and medical history

Patient	Sex	Age (years)	Date of PCR for SARS-CoV-2 (month/day/ year)	Personal History of the patients	Medical History of the patients
**1**	Male	78	02/10/2021	Hypertension treated with olmesartan<br>Aneurysm of the ascending aorta<br>Gout on colchicine<br>Past tuberculosis > 30 y<br>History of smoking (cessation 7 years ago) 20py (pack-year)	Patient 1 presented on 02/10/2021 with fever up to 39°C, dry cough, diarrhea. He was admitted to the hospital due to progressive dyspnea and chest tightness. Laboratory findings upon admission revealed low lymphocyte count, elevated lactate dehydrogenase (LDH), ferritin, C-reactive protein (CRP), and D-dimer. Chest CT findings were bilateral ground-glass opacities and traction bronchiectasis. Before hospital admission he received the azithromycin, doxycycline, and methylprednisolone for 3 days, whereas after hospitalization he was treated with antiviral remdesivir, levofloxacin, dexamethasone and low molecular weight heparin (LMWH).
**2**	Male	45	03/06/2021	Hypertension treated with amlodipine	Patient 2 presented on 03/06/2021 with mild fever up to 37,6°C, dry cough, and diarrhea for the past 15 days for which he was prescribed amoxicillin. He was admitted due to mild hypoxemia and acute respiratory alkalosis. Laboratory findings upon admission revealed elevated LDH, ferritin, and D-dimer. Chest CT findings were bilateral consolidative pulmonary opacities mainly in the lower lobes with focal traction bronchiectasis. The patient was treated with remdesivir, dexamethasone and LMWH. He also received antibiotic treatment with ciprofloxacin and metronidazole.
**3**	Female	63	02/23/2021	Diabetes mellitus type 2 treated with gliclazide, metformin and dapagliflozin.	Patient 3 presented on 02/27/2021 with fever and dyspnea for the past 4 days. She was admitted due to progressive hypoxemia. Laboratory findings upon admission revealed high levels of LDH and ferritin and borderline high D-dimer. Chest CT findings were

				Hypertension on nebivolol and losartan<br>Abdominal Obesity<br>Allergic in amoxicillin, and non-steroidal anti-inflammatory drugs (NSAIDs).	bilateral ground-glass and consolidative pulmonary opacities and emphysematous cysts on the right upper lobe and left lower lobe, compatible with COVID-19 pneumonia. The patient received remdesivir, moxifloxacin, dexamethasone and LMWH.
**4**	Male	56	02/06/2021	Free	Patient 4 presented on 02/25/2021 with dyspnea, progressive oxygen desaturation, and swelling of the left leg for 4 days. He reported fever up to 39°C for 12 days before admission and received antibiotic treatment with azithromycin and cefditoren. He was admitted due to moderate hypoxemia with acute respiratory alkalosis and possible deep vein thrombosis of the left leg. Laboratory findings upon admission revealed neutrophilia, high levels of liver enzymes, LDH, ferritin, D-dimer, and troponin T. Chest CT findings were compatible with COVID-19 pneumonia (bilateral ground-glass and consolidative pulmonary opacities), bilateral pulmonary emboli, spontaneous pneumothorax of the right lung and pneumomediastinum. The patient received LMWH, dexamethasone and levofloxacin. He was not treated with remdesivir due to impaired liver function tests.
**5**	Female	68	02/20/2021	Temporal /Giant-cell arteritis treated with methotrexate and methylprednisolone<br>Smoking 30py	Patient 5 presented on 02/20/2021 with fever up to 39°C for 3 days before admission and received antibiotic treatment with azithromycin. She was admitted due to mild hypoxemia with acute respiratory alkalosis. Laboratory findings upon admission revealed lymphocytopenia and thrombocytopenia, high levels of LDH, CK (creatine kinase), ferritin, and D-dimer. Chest CT findings were compatible with COVID-19 pneumonia (bilateral ground-glass and consolidative pulmonary opacities) and hilar and mediastinal lymphadenopathy. During hospitalization she

					was treated with remdesivir, LMWH, dexamethasone and piperacillin/tazobactam. PCR test on 3/17/2021 was negative.
**6**	Female	70	02/16/2021	Hypertension treated with nebivolol and irbesartan.	Patient 6 presented on 02/22/2021 with type 1 (hypoxemic) - respiratory failure. She reported experiencing fever up to 39°C, myalgia, fatigue, and diarrhea for 7 days before admission. Outpatient management included azithromycin and dexamethasone. Laboratory findings upon admission revealed lymphocytopenia and neutrophilia, high levels of LDH, and D-dimer. Chest CT findings were compatible with COVID-19 pneumonia. During hospitalization she was treated with remdesivir, LMWH, dexamethasone and ceftriaxone.
**7**	Male	52	03/01/2021	Liver abscess due to Klebsiella 3 years ago<br>Antral gastritis	Patient 7 presented on 03/04/2021 with low grade fever up to 38°C, loss of taste, dry cough, and chest tightness for 9 days before admission. He was admitted due to mild hypoxemia. Laboratory findings upon admission revealed high levels of LDH, ferritin, and CK. Chest CT revealed bilateral ground glass opacification. The patient received remdesivir, LMWH, and dexamethasone. PCR test on 3/17/2021 was positive.
**8**	Male	76	03/08/2021	Hyperlipidemia on simvastatin.<br>Diabetes mellitus type 2 treated with metformin.<br>Deforming arthritis on methotrexate.	Patient 8 presented on 03/11/2021 complaining of fatigue and sore throat for 2 days and was admitted due to mild hypoxemia. Laboratory examination revealed high levels of LDH, ferritin, and D-dimer. Chest CT findings were ground-glass opacities and emphysematous cysts of the right lung, compatible with COVID-19 pneumonia. The patient during hospitalization received remdesivir, LMWH, and dexamethasone. PCR test on 3/25/2021 was negative.
**9**	Male	65	12/08/2020	Severe coronary artery disease treated with atorvastatin, eplerenone, torasemide, carvedilol, sacubitril/ valsartan, ivabradine, and acetylsalicylic acid.	Patient 9 presented on 03/10/2021 with type 1 (hypoxemic) - respiratory failure, complaining of fever up to 39°C, myalgia, and fatigue for 7 days, dyspnea and oxygen desaturation, accompanied by a presyncope episode 12 hours before admission. He was treated at home with azithromycin and amoxicillin /clavulanic without any improvement. During hospitalization he received

					remdesivir, LMWH, and dexamethasone. Laboratory findings upon admission revealed lymphocytopenia, high levels of LDH, CK, ferritin, CRP, and D-dimer. Chest CT revealed diffused ground-glass pulmonary opacities and pleural effusion bilaterally. PCR test on 3/25/2021 was negative.
**10**	Female	41	02/22/2021	Treatment of uterine fibroids with GnRH analogue	Patient 10 presented on 03/05/2021 complaining of fever, cough, and progressive dyspnea with oxygen desaturation (SaO2, 77%) a few days before admission. She was admitted due to moderate hypoxemia. Laboratory findings were within the normal reference range. Chest CT was compatible with COVID-19 pneumonia, revealing multifocal ground-glass pulmonary opacities and consolidation in the lower lobes bilaterally. She received remdesivir, LMWH, dexamethasone, and tocilizumab. PCR test on 3/21/2021 was positive
**11**	Male	72	02/11/2021	Free	Patient 11 presented on 02/22/2021 with fever up to 39oC, accompanied by fatigue for 12 days, and progressive dyspnea. He was admitted due to moderate hypoxemia and findings on the CT scan compatible with COVID-19 pneumonia (diffused ground-glass and consolidative pulmonary opacities bilaterally). He was treated with remdesivir, LMWH, dexamethasone, and tocilizumab. PCR test on 3/11/2021 was positive.
**12**	Female	80	03/05/2021	Breast cancer, diagnosed in 2012, treated with ongectomy and anastrozole.<br>Polyarthritis<br>Gastric lymphoma	Patient 12 presented on 03/06/2021 with fever up to 39oC for 3 days. Laboratory findings upon admission revealed thrombopenia, high levels of LDH, CK, ferritin, and D-dimer. Chest CT revealed consolidation in the left upper lobe with traction bronchiectasis, ground-glass opacities in the right lower lobe, and bronchiectasis in the lower lobes bilaterally. She was admitted due to COVID-19 pneumonia and superficial vein thrombosis of the left arm. She received remdesivir and LMWH. During hospitalization she suffered from reactivation of herpes zoster, for which she was treated with valacyclovir. PCR test on 3/16/2021 was positive.

**13**	Female	40	02/23/2021	Free	Patient 13 presented on 03/06/2021 with fever up to 38°C and dry cough for 23 days. She was treated with azithromycin and moxifloxacin in outpatient basis. She was admitted due to hypoxemia. Laboratory findings revealed high levels of LDH. Chest CT revealed multifocal ground-glass opacities, site of consolidation bilaterally, and traction bronchiectasis. She was treated with remdesivir, LMWH, and dexamethasone.
**14**	Male	61	03/05/2021	Hypertension treated with olmesartan medoxomil / amlodipine.<br>Polycythemia Vera on hydroxyurea and acetylsalicylic acid.	Patient 14 presented on 03/24/2021 with myalgia, fatigue, and diarrhea for 10 days, dry cough, chest tightness, and episodes of oxygen desaturation 48 hours prior admission. He was already receiving in outpatient basis colchicine, azithromycin, dexamethasone, and LMWH. During hospitalization he received remdesivir starting on 03/11/2021, moxifloxacin, LMWH, dexamethasone, and tocilizumab. On admission, laboratory testing revealed lymphocytopenia, neutrophilia, elevated LDH, ferritin, and CRP. Chest CT revealed COVID -19 pneumonia bilaterally with diffused ground glass appearance and consolidative pulmonary opacities.
